# Features of Thermal Stabilization of PVC Modified with Microstructured Titanium Phosphate

**DOI:** 10.3390/polym17152140

**Published:** 2025-08-05

**Authors:** Irina N. Vikhareva, Anton Abramian, Dragan Manojlović, Oleg Bol’shakov

**Affiliations:** 1 Nanotechnology REC, South Ural State University, Lenin Prospect 76, 454080 Chelyabinsk, Russia; abramianad@susu.ru (A.A.); bolshakovoi@susu.ru (O.B.); 2 Faculty of Chemistry, University of Belgrade, Studentski Trg 12–16, 11000 Belgrade, Serbia; manojlo@chem.bg.ac.rs; 3 Department for Ecology and Chemical Technology, South Ural State University, Lenin Prospect 76, 454080 Chelyabinsk, Russia; 4 N.D. Zelinsky Institute of Organic Chemistry Russian Academy of Sciences, Leninsky Prospekt 47, 119991 Moscow, Russia

**Keywords:** poly(vinyl chloride), thermal stability, titanium phosphate, hierarchical structure, particle morphology, dehydrochlorination, barrier effect, stabilizer

## Abstract

Poly(vinyl chloride) (PVC) undergoes thermal degradation during processing and operation, which necessitates the use of effective thermal stabilizers. The purpose of this work is to comprehensively evaluate the potential of new hierarchically structured titanium phosphates (TiP) with controlled morphology as thermal stabilizers of plasticized PVC, focusing on the effect of morphology and Ti/P ratio on their stabilizing efficiency. The thermal stability of the compositions was studied by thermogravimetric analysis (TGA) in both inert (Ar) and oxidizing (air) atmospheres. The effect of TiP concentration and its synergy with industrial stabilizers was analyzed. An assessment of the key degradation parameters is given: the temperature of degradation onset, the rate of decomposition, exothermic effects, and the carbon residue yield. In an inert environment, TiPMSI/TiPMSII microspheres demonstrated an optimal balance by increasing the temperature of degradation onset and the residual yield while suppressing the rate of decomposition. In an oxidizing environment, TiPR rods and TiPMSII microspheres provided maximum stability, enhancing resistance to degradation onset and reducing the degradation rate by 10–15%. Key factors of effectiveness include ordered morphology (spheres, rods); the Ti-deficient Ti/P ratio (~0.86), which enhances HCl binding; and crystallinity. The stabilization mechanism of titanium phosphates is attributed to their high affinity for hydrogen chloride (HCl), which catalyzes PVC chain scission, a catalyst for the destruction of the PVC chain. The unique microstructure of titanium phosphate provides a high specific surface area and, as a result, greater activity in the HCl neutralization reaction. The formation of a sol–phosphate framework creates a barrier to heat and oxygen. An additional contribution comes from the inhibition of oxidative processes and the possible interaction with unstable chlorallyl groups in PVC macromolecules. Thus, hierarchically structured titanium phosphates have shown high potential as multifunctional PVC thermostabilizers for modern polymer materials. Potential applications include the development of environmentally friendly PVC formulations with partial or complete replacement of toxic stabilizers, the optimization of thermal stabilization for products used in aggressive environments, and the use of hierarchical TiP structures in flame-resistant and halogen-free PVC-based compositions.

## 1. Introduction

Polymers are the basis of modern industry due to their universal properties: light weight, strength, chemical resistance, and low cost. For this reason, the volume of production and consumption of these materials is constantly increasing [1,2]. Despite discussions about the environmental aspects of the life cycle, especially those related to certain additives and recycling, PVC remains highly relevant due to its unsurpassed functional characteristics, cost-effectiveness, and long service life in key industries such as construction and infrastructure, medicine, and electrical engineering. Continuous improvement of production and processing technologies, as well as the development of safer additives, aims to increase its sustainability and compliance with modern environmental requirements [3,4]. However, during heat treatment and processing of PVC, the problem of thermal instability arises due to the release of hydrogen chloride (HCl) and the rupture of carbon–carbon bonds, which leads to degradation of the material and deterioration in product quality [5,6]. Modification of the polymer using additives opens up wide opportunities for targeted regulation of the technological and operational characteristics of materials and products based on it [7,8]. The decision regarding the type and volume of additives is made taking into account the processing parameters of the polymer composition and the desired set of material characteristics corresponding to the purpose in a specific area of application [8,9,10,11]. In flexible PVC compounds, the main additive used is a plasticizer [12,13]. The use of plasticizers in PVC formulations is necessary to expand the temperature range of polymer processing and impart elastic properties [14,15,16].

When introducing plasticizers into PVC compositions, the thermal stability of the material is significantly reduced. To ensure the stability and durability of PVC products, mandatory thermal stabilization is necessary. Modern thermal stabilizers, such as carboxylates, metal naphthenates, and organic derivatives of azophenol, effectively slow down thermal decomposition, improving the technological and operational characteristics of PVC [8,11].

The main tasks of thermal stabilizers are the substitution of unstable chlorine atoms in the polymer chain to prevent their removal and the formation of defects; absorption and neutralization of released hydrogen chloride; and inhibition of radical chain reactions [17,18].

Currently, a major direction of the development of additives for polymeric materials is the creation of low-toxic and environmentally friendly compounds [19,20,21,22,23]. Heavy metal cations (lead, cadmium) provide high thermal stability but are toxic, which limits their use. At the same time, alkaline earth metals are more environmentally friendly, but require a combination with other stabilizers to achieve an optimal effect.

Titanium phosphates attract researchers with their structure and unique properties, such as high whiteness, light resistance, ion-exchange capacity, and catalytic activity, and are widely used, including in composites with specific properties [24,25]. With sufficiently high pigment properties, titanium phosphate is resistant to high temperatures and aggressive environments and has advantages from the point of view of environmental safety [26,27]. For example, modified amorphous titanium phosphate in composites provides anti-corrosion protection and resistance to thermal and chemical influences [28,29,30].

Materials with a hierarchical structure are of considerable interest due to the unique combination of characteristics of micro- and nanosized objects. Such structures have properties that differ from the properties of their monomorphological analogs and often demonstrate synergistic effects. These materials are characterized by high crystallinity, a particle size of 20–100 nm, and a developed surface of more than 60 m^2^/g (the BET multilayer adsorption method), which ensures the effectiveness of their action in various directions. For practical application in polymers, nanosized materials (nanoparticles, nanotubes, nanoplates) have a number of significant disadvantages: a tendency for aggregation and agglomeration; high energy costs and complexity of the processes of their production; potential migration; and concerns associated with handling nanopowders.

One of the promising solutions is the targeted synthesis of microstructured systems assembled from nanosized building blocks (nanoplates, nanotubes, nanocrystals). This approach allows for obtaining a material that combines a high specific surface area and reactivity of nanoobjects with ease of processing, improved dispersibility, and a reduced tendency for agglomeration, characteristic of microsized particles.

Titanium phosphates stand out among the most promising inorganic materials for creating hierarchical structures. Titanium phosphate nanospheres (TiP) demonstrate high thermal stability, which makes them promising for use as stabilizers of poly(vinyl chloride) (PVC). For example, in [31], titanium phosphates form hierarchical porous structures with micro-, meso-, and macropores that maintain integrity at temperatures up to 500–800 °C. TiP-based materials retain their catalytic activity and structural stability even after prolonged exposure to high temperatures.

The authors note that, unlike organometallic carboxylate carbamides, HPTPs have high hydrolytic stability due to their ability for multimodal coordination and strong Ti–O–P coupling [32]. Hierarchically porous titanium phosphonates (HPTPs) are a new class of hybrid materials that are characterized by structural flexibility, chemical strength, and versatility.

TiP nanoparticles can be dispersed in PVC, creating barrier layers that prevent thermal degradation of the polymer. Their high specific surface area and chemical inertia contribute to the effective absorption of free radicals. Studies have shown that the introduction of TiP into PVC increases its resistance to thermal and oxidative processes. For example, TiP samples retain their mechanical properties when heated to 200 °C, whereas pure PVC degrades already at 150 °C [33].

For effective dispersion of TiP in PVC, surface modifiers such as alkylamines or organophosphates are needed [34]. TiP modification by introducing metals (Cu, Zn) or polymers (e.g., polyaniline) enhances their adsorption properties and thermal stability.

Titanium phosphate nanospheres are a promising class of materials for the stabilization of PVC due to their high thermal stability, chemical inertia, and the possibility of modification. In this paper, it is proposed to use hierarchically microstructured titanium phosphate as an effective stabilizer for poly(vinyl chloride).

Numerous applications of hierarchical structures contributed to the formation of methods for modifying titanium phosphate [35] and the study of the influence of structures on product characteristics. Hierarchical structures of titanium phosphate provide their unique properties due to a complex multi-scale organization, including 0-, 1-, 2-, and 3-dimensional particles and more complex regular structures. Such hierarchy forms a developed surface and a large number of active centers, which enhances the catalytic activity and sorption properties of materials; increased strength and improved conductivity, due to the ordering and interconnection of structural elements of different dimensions; special reaction centers at the phase boundaries, which increase the efficiency of photocatalytic and other reactions [36,37,38,39]. Controlled growth of the oxide phase and surface modification make it possible to create composites with improved functional properties [40]. The main areas of work for use in PVC compositions are the following:

− Morphological control. Mesoporous spheres/dendrites > unstructured powders: specific surface area > 250 m^2^/g is critical for HCl binding;
− Modification strategies. Covalent functionalization (amino-, phospho-no-groups): Ion exchange (La^3+^, Ce^4+^) to enhance acidity, hybridization with GO, TiO_2_, bio-molecules;
− Multifunctionality. Simultaneous improvement of thermal, UV, fire resistance and preservation/improvement of mechanical properties;
− Stability. Focus on non-toxic systems (bio-modifiers, rare-earth ions).

In this regard, the influence of the structure, properties, and quantity of new hierarchically structured titanium phosphates on the thermal stability of plasticized PVC under inert and oxidizing atmospheres was studied. The novelty of the work is to establish morphology as a determining factor of the effectiveness of TiP stabilizers and to prove their synergy with industrial systems. The results open the way to the creation of eco-friendly PVC formulations with controlled thermal and oxidative properties.

## 2. Materials and Methods

### 2.1. Materials

Industrial samples of suspension poly(vinyl chloride) supplied by Bashkir Soda Company Caustic JSC (Sterlitamak, Russia) were used as a matrix. Plasticizer dioctyl phthalate (DOP) was supplied by JSC Kamtex-Khimprom (Perm, Russia). Its main characteristics are acid number (mg KOH/g) ≤ 0.07, saponification number (mg KOH/g) ≤ 289, mass fraction of volatile substances (%, no more) 0.1, flash point (°C) ≥ 205. The stabilizer tribasic lead sulfate (TBLS) is manufactured by Baerlocher GmbH (Ingolstadt, Germany). Appearance: powder, lead content (%)–89; pH–7.0–7.5; ash content (%)—at least 95. The stabilizer calcium stearic acid (CaSt) was produced in the HIMSTAB company (Mytishchi, Russia). Main characteristics: acid number (mg KOH/g)—no more than 2; calcium content (%)—in the range of 6.4–7.5; calcium oxide content (%)—in the range of 9.0–10.5. DL-mandelic acid is manufactured by BingoSpa (Warsaw, Poland), base substance—100%; NH_4_OH is manufactured by NevaReaktiv (Saint Petersburg, Russia), base substance—25%; H_2_O_2_ is manufactured by BiokhimReagent (Ufa, Russia), base substance—40%; titanium powder PTM–1, H_3_PO_4_ is manufactured by Vekton (Saint Petersburg, Russia), base substance—98%; were used without preliminary purification.

### 2.2. Synthesis Methods

Synthesis of the titanium complex with mandelic acid: In accordance with the previously published method [41], 0.41 g (8.5 mmol) of titanium, 40 mL of 40% H_2_O_2_ solution, and 5 mL of NH_4_OH solution are placed in a 100 mL Erlenmeyer flask. The reaction mixture is stirred on a magnetic stirrer until titanium is completely dissolved (from 1 to 1.5 h), maintaining the temperature of the mixture in the range of 5 to 10 °C. After dissolution, a light yellow solution of titanium peroxocomplex is filtered and an organic acid (17 mmol) solution in 2–3 mL of water is added to it. After adding an acid, the temperature of the reaction mixture is kept between 15 and 20 °C for another 2–3 h. Isolation of titanium complexes with hydroxoacids from aqueous solutions is carried out by evaporation at a reduced pressure in a rotovap at 30–40 °C.

Obtaining microstructured titanium phosphate (TP) via the hydrothermal method: A water-soluble titanium complex with DL-mandelic acid (1.66 mmol) dissolved in 5 mL of water was placed in a Teflon-coated autoclave, after which an appropriate amount of phosphoric acid was added to the complex. The resulting white cloudy solution was diluted with water to 20 mL. The prepared solutions were sealed and heated. After cooling the autoclave, the solid particles were separated from the solutions by washing and centrifugation.

### 2.3. Characterization Microstructured TP

The registration of IR transmission spectra was carried out on a Shimadzu IRAffinity S1 IR-Fourier spectrometer (Kyoto, Japan) in the range from 400 to 4000 cm^−1^ with a resolution of 4 cm^−1^ and in the number of 40 repetitions. Elemental analysis and the morphology of the samples were studied using a Jeol JSM 7001F electron microscope (Akishima, Japan) equipped with an Oxford INCA X-max 80 energy dispersive spectrometer (Abingdon, UK). The accelerating voltage of the electron gun was set to 20 kV, as required for quantitative EDS analysis. The TEM microphotographs (Dallas, TX, USA) of TP were recorded with a transmission electron microscope JEOL JEM-2100 (Akishima, Japan) with a working voltage of 200 kV. The phase composition and structure of the samples were studied on a Rigaku Optima IV powder diffractometer (Akishima, Japan). The survey was carried out in the range of 2θ angles from 5° to 90° at a survey rate of 5°/min. The study used radiation from a CuKα copper tube (λ = 1.541 Å) at an accelerating voltage of 40 kV. Specific surface area and porosity were measured by low-temperature nitrogen adsorption on an ASAP Micromeritics 2020 instrument. The measurement stage was preceded by a degassing carried out at a temperature of 200 °C for 2 h. The specific surface area was determined based on the BET multilayer adsorption method. The volume of mesopores and their size distribution was calculated based on the Barrett–Joyner–Halenda (BJH) model.

### 2.4. Preparation of Prototypes Containing Microstructured TP

**Figure 1:**
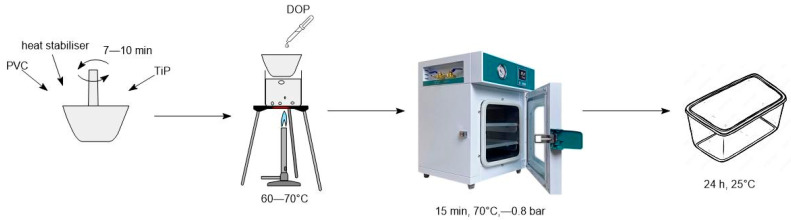
Scheme of preparation of plasticized PVC samples.

The PVC resin samples were mixed with the required amount of heat stabilizer for 7–10 min using circular motions. Then, the plasticizer was added. The mixture was heated in a water bath at 60–70 °C to improve dispersion. The PVC composition was transferred to a vacuum chamber and maintained at –0.8 bar and 70 °C for 15 min to remove trapped air. Afterwards, the mixture was transferred to a container and kept at 25 °C for 24 h to ensure complete homogenization of the system (Figure 1).

### 2.5. Methods of Analysis

Determination of thermal stability of polymer compositions based on poly(vinyl chloride) by the TG method.

The analysis was performed using the Netzsch STA 449 F1 Jupiter thermal analysis device (Selb, Germany), which is a combined thermogravimetric analysis device and differential scanning calorimetry. The temperature range of the device is 25–1650 °C. The maximum sample heating rate is 50 K/min, and the cooling rate of the device is 20 K/min. The measurement error is ± 0.3 K. Thermal analysis of the samples was carried out over the temperature range of 25 to 600 °C in either an argon or air environment. Measurements were conducted in dynamic mode at a constant heating rate of 10 °C/min. The sample mass for measurements was 5–15 mg. Pt/Rh crucibles were used. Data processing was performed using a computer. During the measurements on the STA thermal analyzer, the resulting thermogram of the sample records curves used to assess its thermal stability:

− The thermogravimetric (TG) curve shows the change in the sample mass during heating (mass vs. temperature graph);
− The differential thermogravimetric (DTG) curve shows the rate of mass change during heating;
− The DSC curve describes thermal effects occurring in the sample during the heating process.

From the obtained curves, the following characteristics were determined to evaluate thermal stability:

− Ts, °C—temperature at which the sample mass begins to decrease during heating;
− Δm200 °C, %—mass loss of the sample when heated to 200 °C;
− Δm5%, °C—temperature corresponding to 5% mass loss;
− Δm10%, °C—temperature corresponding to 10% mass loss;
− T1max DTG, °C—temperature of the first maximum on the DTG curve, indicating the maximum rate of product decomposition (first peak);
− T2max DTG, °C—temperature of the second maximum on the DTG curve, indicating another decomposition rate peak (second peak);
− Tmax DSC, °C—temperature of the maximum endothermic peak corresponding to the main dehydrochlorination stage on the DSC curve;
− ΔCp, J/(g·K)—change in heat capacity during the key structural transition in the initial compound destruction phase, mainly linked to dehydrochlorination;
− ΔH, J/g—enthalpy of the main dehydrochlorination stage, calculated by integrating the peak area on the DSC curve and considering the calorimetric constant of the device.

To ensure accuracy, controlling experimental conditions and accounting for the plasticizer’s effect on polymer thermal stability are essential.

## 3. Results

Earlier works described the production and study of possible applications of hierarchically structured titanium phosphates [24,35,41]. The main characteristics of the titanium phosphates used in the work are given in Table 1.

**Figure 2:**
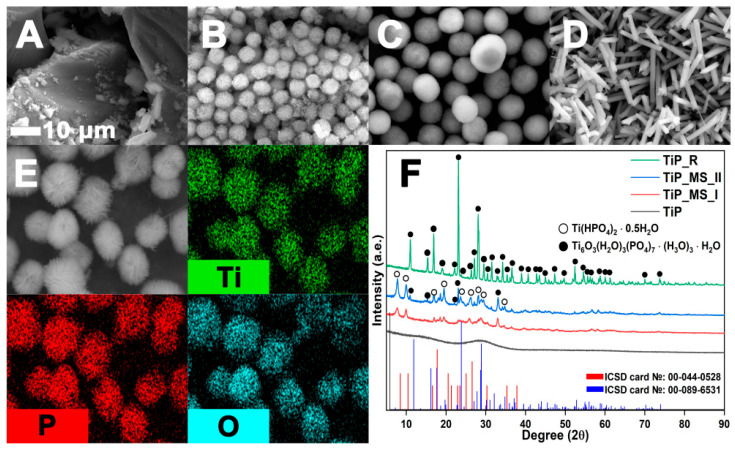
(**A**–**D**) SEM images of TiP samples, TiPMSI, TiPMSII microspheres, and TiPR hexagonal microrods; (**E**) EDS analysis; (**F**) X-ray diffraction analysis.

In brief, the hydrothermal synthesis products exhibit three types of morphology: amorphous precipitate (TiP), microspheres (TiPMSI, TiPMSII), and hexagonal microrods (TiPR) (Figure 2A–D).

Amorphous titanium phosphate (TiP) is formed by short-term synthesis with a PA:Ti molar ratio ≥1:4. A decrease in the PA concentration prevents the formation of a precipitate (Figure 2A). Microspheres are formed in a wide range of parameters, and under strict control of conditions, they acquire a regular shape with a narrow size distribution. Their structure is a spherically ordered aggregate of microflakes (Figure 2B,C). Long-term synthesis (≥48 h) leads to the recrystallization of microspheres into thermodynamically stable hexagonal TiPR rods (Figure 2D). EDS analysis confirms a uniform distribution of Ti, P, and O elements (Figure 2E). X-ray diffraction analysis revealed the evolution of the composition from amorphous TiP (4 h) through the two-phase system Ti(HPO_4_)_2_•0.5H_2_O/Ti_6_O_3_(H_2_O)_3_(PO_4_)_7_•(H_3_O)_3_•H_2_O (12 h) to the single-crystalline phase Ti_6_O_3_(H_2_O)_3_(PO_4_)_7_•(H_3_O)_3_•H_2_O (≥48 h) (Figure 2F; ICSD Nos. 00-044-0528 and 00-089-6531).

To study the effect of hierarchically structured titanium phosphates on the thermal stability of PVC, samples of PVC compounds with different stabilizer contents and the resulting hierarchically structured titanium phosphates were obtained. Then, the samples were studied by thermogravimetry in an inert (Ar) and oxidizing (air) atmosphere.

Initially, the effect of additives in the formulation of a PVC composition of the following composition was studied in an inert (Ar) atmosphere, mass parts: PVC S-7059M–100; plasticizer DOP–100; calcium stearate–5, Ti phosphate–1 (Table 2). PVC-p is a basic PVC composition without titanium phosphate content.

**Figure 3:**
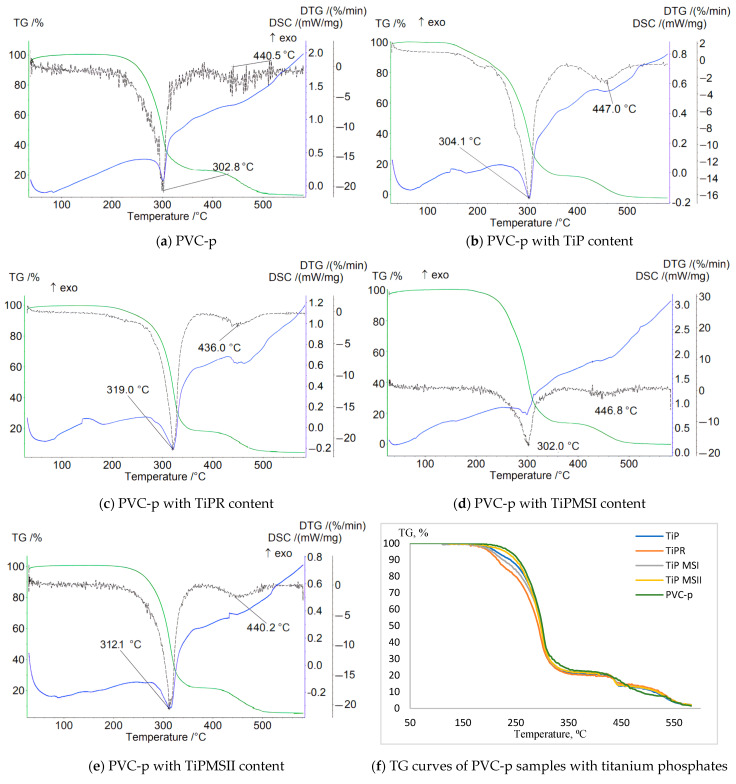
TGA thermograms of PVC-p samples in an Ar atmosphere.

Data characterizing the mass loss of samples in an inert atmosphere revealed a significant increase in the thermal stability of PVC composites containing TiPR and TiPMSII (Table 2). This is evidenced by a shift of the T1max DTG peak to higher temperatures—319 °C and 312.1 °C, respectively—compared to 302.7 °C for the plasticized base PVC sample without titanium phosphate. The sample containing TiPMSII exhibited the lowest weight loss up to 200 °C—only 0.5%—and the highest onset temperature of thermal degradation at 167 °C, compared to 162 °C for the base formulation. The Δm5% and Δm10% values were also the highest among the studied titanium phosphate modifications (Figure 3). Although the onset temperature (Ts) decreased for the PVC composition with the TiPR additive, the maximum peak on the DTG curve for this sample reached 319 °C, substantially higher than that of the base composition and samples containing other titanium phosphate modifications.

For all samples, the obtained thermograms show stepwise degradation with a high residual mass at 581 °C, indicating high thermal stability of the carbonized residue stabilized by titanium phosphate (Figure 3).

The DTG curve (derivative of the TG curve) reveals three main stages of degradation, corresponding to different phases of the composition’s decomposition:

Stage 1 corresponds to the onset of intensive PVC dehydrochlorination (HCl cleavage) and the beginning of evaporation and decomposition of the DOP plasticizer. The mass loss at this stage is minimal (marked by the start of a steep decline on the TG curve).

Stage 2, the main peak, is associated with the maximum rate of PVC dehydrochlorination and active decomposition of the DOP plasticizer. The majority of mass loss occurs during this stage.

Stage 3 corresponds to the breakdown of the unsaturated carbon skeleton of the polymer polyene structure formed during dehydrochlorination and the final decomposition of DOP.

The stabilizer used in the base PVC composition, calcium stearate, effectively slows the initial dehydrochlorination; the DTG peak is shifted to higher temperatures compared to pure unstabilized PVC (287 °C), and it promotes the formation of a thermally stable carbon residue.

The TG curve for the sample containing amorphous TiP demonstrates a smooth weight decrease with the lowest onset temperature of weight loss and the greatest weight loss up to 200 °C. The sharpest weight decreases correspond to samples with TiPMSI and TiPMSII microspheres. The onset temperatures of weight loss for these samples are the highest, and they also correspond to minimal weight losses up to 200 °C. However, the large microspheres in the TiPMSII sample, which contain more phosphorus, more effectively increase the thermal stability of the PVC composite. All titanium phosphates significantly enhance the thermal stability of the PVC composition compared to the base formulation, reflected on the TG curve by an increase in the onset temperature of decomposition (especially for TiPMSI and TiPMSII); an increase in residual mass at 580–600 °C; and suppression of the decomposition onset for TiPMSI and TiPMSII.

On the DTG curve, titanium phosphates in the PVC composition alter the degradation pattern: they shift the degradation peaks to higher temperatures (notably TiPMSII and TiPR); they significantly reduce the maximum rate of mass loss (DTG peak intensity), except for TiP, indicating a slowdown in degradation processes. Thus, two degradation stages are clearly visible for the base PVC, while for compositions with phosphates, the peaks often merge or shift, indicating a change in the degradation mechanism and kinetics under their influence. The most pronounced suppression of the degradation rate is observed for TiPMSI and TiPMSII.

The DSC curves of PVC samples containing modified titanium phosphates exhibit a more complex profile and a greater number of peaks than the base compound. For all samples with the studied additives, clear peaks appear below 200 °C, as well as an endothermic peak corresponding to the greatest mass loss on the TG curve, and additional peaks above 400 °C corresponding to high-temperature decomposition/carbonization of the thermally stable carbonaceous residue formed after the main stages of PVC and DOP decomposition. The endothermic nature of these peaks confirms pyrolytic decomposition with energy absorption.

All titanium phosphates significantly suppress the exothermic processes accompanying the initial stages of PVC decomposition (dehydrochlorination). The release of HCl catalyzes autocatalytic degradation. Titanium phosphates (especially TiPMSII) suppress the exothermic effect by binding HCl and providing a barrier effect (increasing the enthalpy ΔH of the endothermic peak). For example, TiPMSII shifts the DSC Tmax to 310.3 °C and increases ΔH to 142 J/g compared to 114 J/g for the base PVC composition. This indicates their effectiveness as heat stabilizers, slowing down unwanted chain reactions; TiPMSII microspheres demonstrate the most effective suppression of the exothermic effect (Table 3).

TMSII microspheres and rods provide greater stability compared to amorphous TiP. TiPR and TiPMSII significantly increase the Tmax of dehydrochlorination. The maximum increase in ΔH in TiPR (203.8 J/g) indicates an increase in endothermal stabilization processes.

The ratio of Ti to P in the studied additives does not have a pronounced effect on the thermal stability of the PVC composition; instead, the samples demonstrate a clear influence of morphology. TiPMSII microspheres exhibited the best overall set of properties. They most effectively increase the onset temperature of decomposition of the PVC composition (particularly TiPMSII) and suppress the exothermic effects of thermolysis (especially TiPMSI). TiPR microrods in the composite showed a very high residue on thermograms and good suppression of the decomposition rate (moderate DTG intensity), but they are inferior to microspheres in raising the decomposition onset temperature and in suppressing exothermic processes. Although the amorphous TiP sediment improves stability compared to the base composition (high residue), it is inferior to structured forms, especially microspheres, in several indicators (onset of decomposition, exothermic intensity. Presumably, titanium phosphates, especially those with a developed structure—microspheres and rods—provide stabilization primarily via adsorption of HCl. By binding hydrogen chloride released during PVC dehydrochlorination, they suppress the autocatalytic nature of this reaction, which explains the shift of the DTG peaks toward higher temperatures (to the right) and the suppression of exothermic peaks on the DSC curves. Additionally, structured particles can create a barrier that hinders the diffusion of volatile decomposition products from the bulk material and/or reduces heat transfer. Ti ions may also interact with unstable chlorine-containing groups in PVC, replacing them and thereby enhancing the stability of the polymer chain. The tables show the effect of additives in an oxidizing atmosphere in the formulation of a PVC composite of the following composition. Mass parts: PVC S-7059M–100; plasticizer DOP–100; calcium stearate–5, phosphate Ti–1 (Table 4 and Table 5).

TiPMSI sharply reduces ΔH (118.6 J/g), which is explained by the catalytic oxidation of the carbon skeleton. The Tmax for all samples is close to the base due to the masking of the endothermic peak by exothermic oxidation.

**Figure 4:**
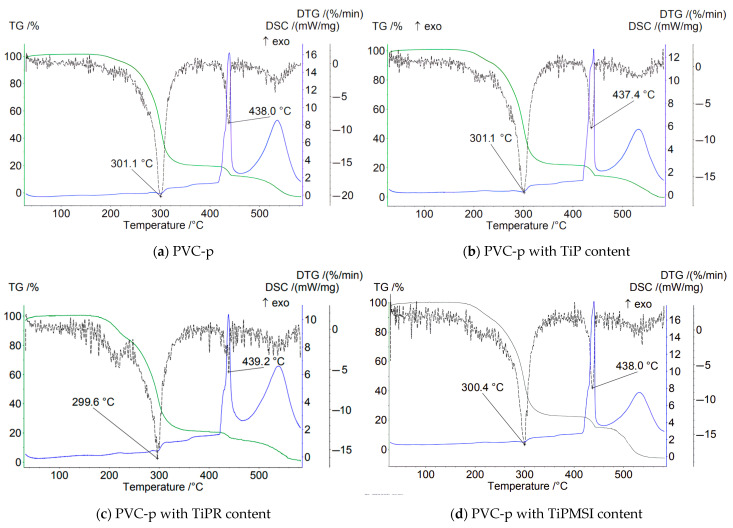

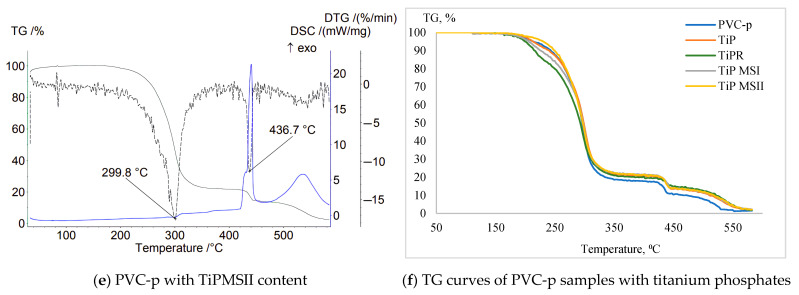
TGA thermograms of PVC samples in an air atmosphere.

In an air atmosphere, i.e., under oxidizing conditions, no significant increase in the thermal stability of PVC compositions is observed when the additive content is 1 part by weight per 100 parts by weight of PVC. On the contrary, the use of these additives results in a shift of the main peak on the DTG curve by 1–3.4 °C toward a lower temperature region compared to the base formulation (Figure 4).

At the stage of PVC dehydrochlorination corresponding to the first step of the TG curve, the maximum loss of sample mass is observed for all samples. The highest temperature of the onset of thermal degradation and a sharp loss of mass in this region are characteristic of the sample containing hexagonal TiPR rods with a high phosphorus content (Ti/P = 0.86), and is 164 °C, which is significantly higher than that of PVC composites with other titanium phosphate modifications and the base formulation. However, TiPR leads to a slight decrease in the Tmax value on the DTG curve. Additionally, the consolidation of the degradation stages of the PVC composition containing TiPR can be attributed to the ordered structure of the additive.

Amorphous TiP and TiPMSI and TiPMSII microspheres in an oxidizing atmosphere shift the main PVC dehydrochlorination peak (Tmax DTG) to higher temperatures compared to other studied samples. Large TiPMSII microspheres provide a better barrier effect than the smaller ones, as evidenced by the high values of 4–5 reported in Table 3. Small TiPMSI microspheres, however, significantly reduce the onset temperature of PVC decomposition to 112 °C.

Thus, in an oxidizing atmosphere, amorphous TiP, which has a disordered structure and a Ti/P ratio of 0.85, exhibits low efficiency in barrier properties for PVC stabilization. The TiPR additive, consisting of crystalline hexagonal rods with a Ti/P ratio of 0.86, offers the maximum shift in the onset temperature (Ts) by +7 °C and demonstrates high barrier efficiency.

Small spherical TiPMSI aggregates with a Ti/P ratio of 0.82 and high surface area exhibit a gradual decrease in sample mass. The protective effect is more pronounced when using large TiPMSII spheres, which may be attributed to their higher titanium content (Ti/P = 0.86).

In an oxidizing atmosphere, modified titanium phosphates neutralize HCl (a product of PVC dehydrochlorination), thereby slowing down autocatalytic degradation. Hierarchical structures—especially microspheres and rods—create a physical barrier that hinders the diffusion of oxygen and volatile decomposition products. The optimal Ti/P ratio of 0.86 (found in TiPMSII) promotes the formation of Ti^4+^ centers that effectively bind HCl. Deviations from the Ti/P stoichiometry reduce the activity of titanium phosphates. Moreover, crystalline phases (such as TiPR and Ti_6_O_3_(H_2_O)_3_(PO_4_)_7_) are thermally more stable than amorphous ones, as confirmed by X-ray diffraction data.

The DSC curves for samples containing the studied additives show no clear minima at the initial stage of dehydrochlorination, despite the presence of peaks in the TG and DTG curves. The main reasons are as follows:

− Overlapping processes: In air, endothermic dehydrochlorination is masked by exothermic oxidation. Oxygen initiates radical reactions. A clear exothermic signal is masked [42,43].
− The catalytic role of Ti^4+^: Titanium ions generate superoxide radicals, accelerating oxidation already at 200–300 °C. This reduces the intensity of the isolated peak of dehydrochlorination [44,45,46,47].

Comparative analysis of thermolysis of samples in oxidizing and inert atmospheres showed the following. In argon, degradation begins at a higher temperature than in air, due to the absence of oxidative processes that accelerate degradation. In an argon atmosphere, the DTG curve for all samples exhibits a single clear peak corresponding to dehydrochlorination. The absence of high-temperature peaks (>300 °C) in argon confirms the suppression of oxidative reactions, whereas in air, these processes are manifested as at least two distinct peaks.

The effect of the structure and composition of amorphous TiP (Ti/P = 0.85) on thermal stability is as follows. At this ratio, the excess titanium content forms Ti^4+^ centers capable of binding HCl; however, their efficiency is lower than that of phosphates with Ti/P ≈ 0.86. The lack of an ordered structure reduces the barrier effect against oxygen diffusion (in air). The mechanism of action differs by atmosphere: in air, TiP slows dehydrochlorination by binding HCl, but oxidation of the hydrocarbon skeleton proceeds actively; in argon, TiP moderately stabilizes PVC but, due to its amorphous nature, does not prevent DOP evaporation at early stages. Thus, TiP exhibits moderate activity in both environments.

The hexagonal rod morphology of TiPR forms a labyrinthine structure within the PVC matrix, hindering the diffusion of both oxygen and volatile decomposition products. The high Ti/P ratio of 0.86 corresponds to titanium deficiency, which in an inert atmosphere facilitates the binding of HCl (a dehydrochlorination product), thereby slowing autocatalytic decomposition. In an oxidizing atmosphere (air), Ti^4+^ ions catalyze oxidation of the carbon skeleton, reducing the temperatures at which mass loss occurs [43,44,45]. Titanium deficiency creates vacancies that adsorb HCl by forming Ti–Cl bonds. The observed shift of Ts to 164 °C in air confirms the inhibitory effect of TiPR during the initial stages of degradation.

Small TiPMSI microspheres slow decomposition in argon via barrier effects and HCl binding, exhibiting a single-stage decomposition behavior. In air, a sharp mass loss occurs after 400 °C due to catalytic oxidation of the carbon skeleton, followed by two-stage combustion of the sample. The hierarchical structure of TiPMSI microspheres (Ti/P = 0.82), with high specific surface area and porosity, enables effective HCl binding, thereby slowing autocatalytic degradation in argon. However, in oxidizing conditions, the same structure catalyzes oxidation due to oxygen transfer to the titanium surface. Consequently, in an inert atmosphere, TiPMSI microspheres act as a physical barrier and “trap” for HCl, increasing the dehydrochlorination peak temperature by +15 °C compared to pure PVC (287 °C). Conversely, in air, titanium ions catalyze radical oxidation of organic fragments, lowering the combustion temperature.

For large TiPMSII microspheres in an inert environment, the initial degradation stage observed on the TG curve (~250–300 °C) corresponds to loss of plasticizer (DOP) and volatile impurities. The main degradation of the PVC composite proceeds in two stages: ~280–350 °C—HCl elimination with formation of double bonds in the polymer chain; ~400–500 °C—rupture of the carbon chain forming low-molecular hydrocarbons. Peaks at 312 °C and 436 °C on the DTG curve confirm this two-stage degradation. TiPMSII shifts these peaks to higher temperatures, indicating enhanced thermal stability.

In an oxidizing atmosphere, degradation begins at lower temperatures (~200–250 °C) due to oxidation. The main degradation stages are: ~250–350 °C—HCl elimination and polymer chain oxidation; ~350–500 °C—complete oxidation of the carbon skeleton producing CO_2_ and H_2_O. Corresponding peaks on the DTG curve at ~300 °C and ~400 °C reflect accelerated oxidation-driven degradation. TiPMSII slows oxidation, as manifested by a decreased rate of weight loss. Its hierarchical structure and large microspheres provide a high specific surface area, enhancing interaction with PVC and retarding degradation. This structure facilitates the formation of a protective layer that prevents the diffusion of volatile products.

The optimal Ti/P ratio of 0.86 further enhances stabilization by effective binding of HCl released during PVC degradation and catalytic formation of a carbonaceous layer, which slows further degradation.

Double maxima are observed in the DSC curves under an air atmosphere at high temperatures (400–500 °C). The first maximum (400–450 °C) corresponds to the oxidation of the polyene skeleton formed after dehydrochlorination. –O_2_•^−^ radicals attack the C=C double bonds [44,48,49]:

Ti^3+^–O_2_•^−^ + >C=C< + 2H^+^ → Ti^4+^ + >C=O + H_2_O + >CH_2_.

The second maximum (450–500 °C) corresponds to the complete oxidation of the carbonaceous residue to CO_2_ and H_2_O, accompanied by the release of energy (exothermic peak).

In an argon atmosphere, the presence of TiPMSII in the PVC composite formulation results in slower degradation with clearly defined stages. Titanium phosphate effectively enhances thermal stability by increasing the degradation temperature. In contrast, accelerated degradation occurs in air due to oxidation. Although titanium phosphate partially mitigates oxidative processes, its stabilizing effect is less pronounced in an oxidizing environment than in an inert one.

To study the effect of additive content on the thermal stability of PVC compounds, composites with the following composition were prepared. Mass parts: PVC S-7059M–100; plasticizer DOP–100; calcium stearate–5, phosphate Ti–5. The results of testing samples in an oxidizing atmosphere are presented in Table 6 and Table 7.

**Figure 5:**
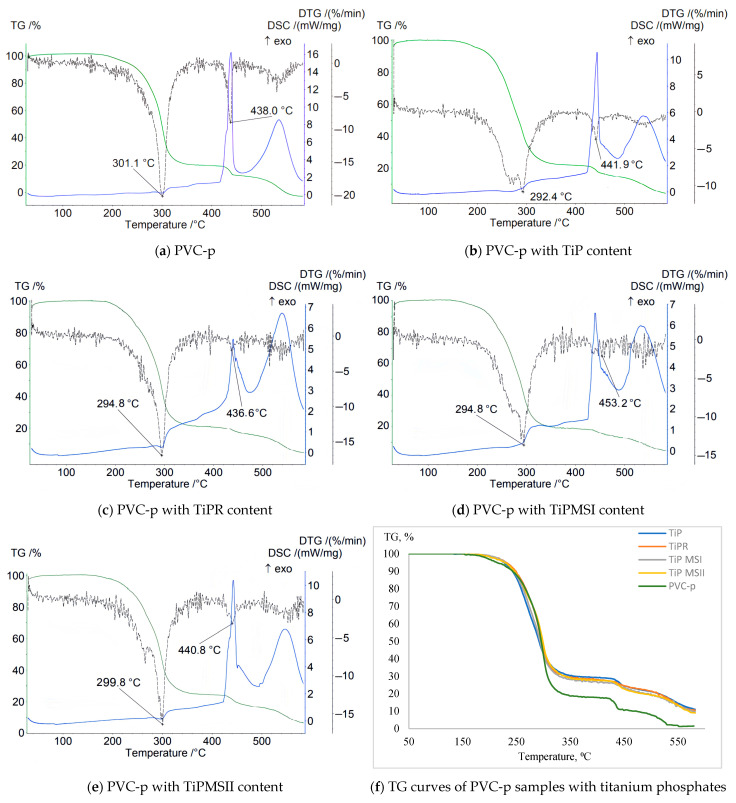
TGA thermograms of PVC samples with a high TiP content in an air atmosphere.

An increase in the amount of titanium phosphate for all studied modifications in compositions using calcium stearate as a stabilizer leads to a shift of the peak on the DTG curve toward lower temperatures (Figure 5). However, compared to the data in Table 4, a significant increase is observed in the following parameters: the onset temperature of thermal degradation of the sample; the temperatures corresponding to 5% and 10% mass loss; and a notable decrease in mass loss at 200 °C. Similar to previous cases, the lowest effectiveness is observed for the titanium phosphate sample TiP in the form of an amorphous sediment. All studied titanium phosphate structures in the PVC compositions contribute to the formation of a protective layer that inhibits the diffusion of oxygen and volatile products. This effect is most pronounced when using hexagonal TiPR rods, which create a labyrinthine structure within the titanium phosphate modification.

Titanium phosphates of all studied modifications reduce the onset temperature (Ts) of PVC decomposition compared to the base composition, except for the TiPR additive, which has Ts = 162 °C. The peak on the TG curve for PVC compositions containing titanium phosphates spans a shorter temperature range, i.e., it is more compressed in time compared to the base composition. The decomposition onset occurs at higher temperatures, while the end occurs at lower temperatures. Consequently, the weight loss at 200 °C for all studied samples is significantly less than that of the base PVC composition.

The DTG curve peak corresponding to the dehydrochlorination stage is considerably broader than that of the base PVC, and for all samples except TiPR, this peak clearly splits into several distinct peaks. Thus, titanium phosphates, with the exception of amorphous TiP, initially exert a barrier effect on the thermal decomposition of PVC, followed by catalytic acceleration of the early decomposition stage as temperature increases.

Although the amorphous TiP precipitate reduces oxidation intensity, its stabilizing effect is weaker than that of structured forms. The higher specific surface area of amorphous TiP may explain its stronger catalytic effect on dehydrochlorination, as reflected by the lowest values of Ts, Δm5%, and Δm10%.

Titanium phosphates effectively suppress the exothermic oxidation of the carbonaceous residue. The oxidation peak for PVC samples containing titanium phosphates (except those with TiPR) shifts to higher temperatures. Furthermore, the oxidation peaks on the DTG curves show significantly reduced intensity (notably for TiPMSII, TiPR, and TiP), demonstrating that titanium phosphates slow and suppress oxidation of the formed char.

The elongated morphology of TiPR likely promotes the formation of a network structure in the residue, creating a more effective barrier that slows oxygen diffusion to the char and inhibits the release of volatile products. Microspheres (TiPMSI, TiPMSII) exhibit a less pronounced effect on dehydrochlorination than microrods but more effectively shift the char oxidation temperature upwards and suppress the exothermic peak.

The Ti/P ratio does not demonstrate a clear correlation with thermal stability or fire protection properties. For example, TiPMSII and TiPR both have a Ti/P ratio of 0.86 but exhibit very similar behavior despite their distinct morphologies. TiP (Ti/P = 0.85) and TiPMSI (Ti/P = 0.82) differ in properties, likely due to morphology rather than stoichiometry.

Within this Ti/P ratio range, particle morphology has a more significant influence on the thermal-oxidative stability of PVC compositions than minor stoichiometric variations.

Thermolysis comparison of PVC composites with varied additive content in air:

PVC composites with increased TiP content exhibit higher initial thermal stability. A delay in the onset of dehydrochlorination by approximately 20 °C indicates improved efficiency of TiP at higher loading in binding the released HCl at early stages. Despite the delayed onset, the maximum rate of dehydrochlorination occurs earlier by about 6.5 °C, possibly because smaller additive amounts less actively catalyze later stages, resulting in a less sharp decomposition peak.

The onset temperature of PVC carbon skeleton oxidation (post-dehydrochlorination) is higher for composites with increased TiP, indicating more effective stabilization of the polyene structure, thus slowing its oxidation and degradation. This stabilization is critically important for fire resistance. The oxidation rate peak on the DTG curve for these composites is also shifted to higher temperatures (~4 °C), reaching approximately 441 °C, consistent with increased char stability.

Regarding the TiP structure and stabilization mechanism, the Ti/P ratio of 0.85 for TiP reflects phosphorus excess, typical for amorphous titanophosphates structurally rich in Ti–O–P bonds. Titanium deficiency causes the suppression of Ti–O–Ti bonds (domination of isolated [TiO_6_] octahedra in the phosphate matrix) and results in a pyrolysis residue with high P_2_O_5_ content. The principal stabilization mechanism is the enhanced binding of HCl (at 200–250 °C) by the main ≡P–O^−^/P=O groups.

PVC compositions containing TiPR display the highest thermal stability of decomposition onset (Ts). The large residue formed upon increasing TiPR content confirms the barrier effect of the phosphate; rod morphology creates a physical shield that slows pyrolysis. For PVC containing one part TiPR by weight, two-stage dehydrochlorination peaks appear at 219 °C and 296.6 °C, whereas the five parts TiPR formulation yields combined peaks at 294.6 °C, indicating slowed degradation due to the rods’ barrier effect.

An increase in TiPR content reduces the decomposition rate, confirming phosphate’s inhibitory role. The rod-like particles produce a labyrinth effect, hindering diffusion of volatile products and oxygen.

Increasing TiPMSI content raises key decomposition temperatures; however, the dehydrochlorination peak shifts to lower temperatures. TiPMSI microspheres form an extremely effective barrier against oxidation of the polyene structure, resulting in high residual mass. Excess phosphate (TiPMSI) reduces this protection at high temperatures, likely due to particle agglomeration or changes in coke structure.

DTG curves show that increasing TiPMSI produces a broad peak for the first degradation stage and multiple overlapping peaks in the ranges 350–385 °C and above 450 °C. The morphology of TiPMSI microspheres stabilizes dehydrochlorination, but increased additive amount complicates high-temperature decomposition/oxidation, introducing multiple faster degradation stages.

Only the sample with the highest TiPMSI content exhibits intense exothermic oxidation of the char in air. This confirms the TG/DTG conclusion that excess additive diminishes the barrier properties of the coke/ash layer, rendering the carbon skeleton more prone to oxidize with heat release.

The Ti/P ratio of 0.82 in TiPMSI indicates a phosphorus-enriched titanium phosphate structure, likely optimal for interaction with PVC and its decomposition products.

On TiPMSII effects: TiPMSII in PVC slows dehydrochlorination through HCl binding. Its effect is enhanced due to higher phosphorus content (Ti/P = 0.86).

Increasing TiPMSII in PVC under an oxidizing atmosphere boosts initial thermal stability, but the differences diminish at higher temperatures.

The DTG peak related to polyene chain decomposition shifts to higher temperatures and decreases in intensity with increasing additive. The phosphate forms a barrier layer that slows oxygen diffusion; higher TiPMSII content leads to a denser protective layer.

DSC data reveal that TiPMSII acts as a thermal barrier, absorbing part of the heat. Its hierarchical microsphere structure compensates for oxidation processes.

The Ti/P ratio of 0.86 corresponds to acidic phosphates (predominance of HPO_4_^2−^/H_2_PO_4_^−^ groups), which actively bind HCl and suppress catalytic PVC decomposition.

Larger amounts of additive shift dehydrochlorination peaks to lower temperatures but simultaneously form a denser protective layer.

In order to study the synergistic effects when used together with other stabilizers, PVC composites of the following composition were obtained. Mass parts: PVC S-7059M–100; plasticizer DOP–100; tribasic lead sulfate (TBLS)–4, calcium stearate–1, phosphate Ti–1. The results of testing the samples in an oxidizing atmosphere are presented in Table 8 and Table 9.

In general, changing the stabilizer composition—i.e., partially replacing calcium stearate with TBLS—while maintaining the same amount of titanium phosphate in an oxidizing atmosphere leads to a significant increase in the parameters Ts, Δm5%, and Δm10%, and a significant decrease in Δm200 °C, for all studied PVC samples. For PVC samples containing TiPMSII and TiPR, a shift of the maximum peak on the DTG curve to a higher temperature region (307.3 and 306.1 °C, respectively) is also observed. Such changes in these indicators undoubtedly confirm an improvement in the thermal stability of PVC compositions with the studied additives when using this stabilizing system.

**Figure 6:**
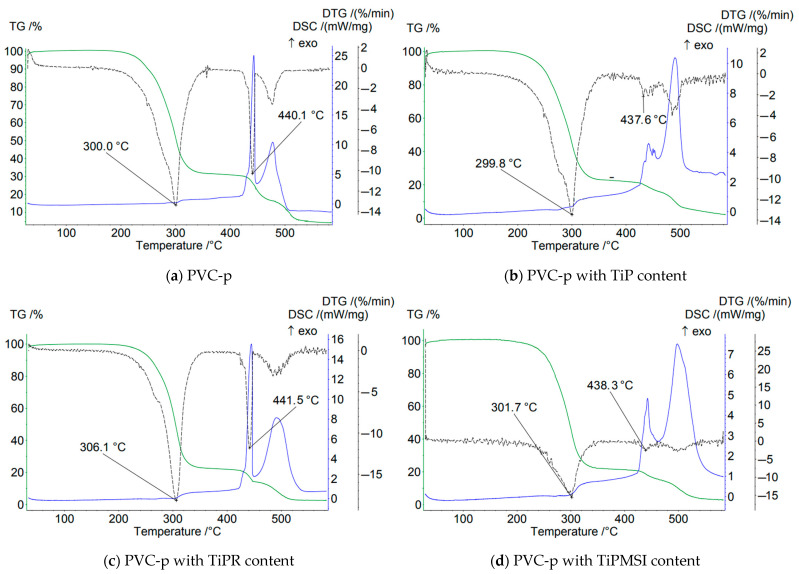

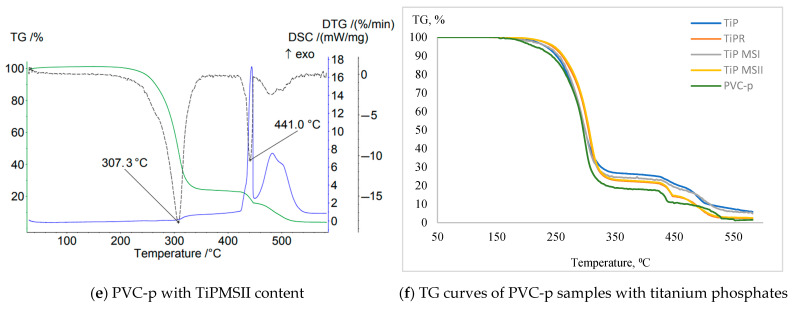
TGA thermograms of PVC samples with an integrated stabilizer in an air atmosphere.

Thus, the TG curves of the base composition show two stages of decomposition: dehydrochlorination and oxidation of the residue (Figure 6). The modified compositions retain these two stages. Notably, compositions with additives of TiPMSI microspheres (Ts + 28 °C), TiPMSII (Ts + 5 °C), and hexagonal rods (Δm5% + 5 °C) are characterized by noticeable shifts toward higher temperature regions. All compositions show an increase in residual carbon; the largest residue at 583 °C is for TiP (5.94%), and the smallest is for TiPR (2.43%). This indicates enhanced carbonization.

Differential thermogravimetry (DTG) shows a shift of the dehydrochlorination peak to higher temperatures by 1.7–7.3 °C for the PVC compositions containing TiPR, TiPMSI, and TiPMSII modifications. TiPMSII (441 °C) and TiPR (441.5 °C) phosphates exhibit the greatest delay in the oxidation peak.

All additives reduce the rate of dehydrochlorination by 10–30%, with TiP showing the most significant effect: −17.97%/min compared to −20.33%/min for the base PVC composition.

On the DSC curves, the temperatures of the exothermic oxidation peaks correlate well with DTG data and are shifted to higher temperatures for the modified samples. Additionally, the peak intensities are decreased, confirming the inhibitory effect of the titanium phosphates on oxidation.

Thus, the amorphous TiP precipitate in the PVC composition, when used with the complex stabilizer calcium stearate/tribasic lead sulfate, provides moderate suppression of the decomposition rate and the highest residual mass; hexagonal TiPR rods result in a pronounced peak shift but the lowest residue; small TiPMSI microspheres offer a maximum barrier effect demonstrated by the largest shift in Ts, strong suppression of the decomposition rate, and high residue; and large TiPMSII microspheres produce the greatest peak shift and high barrier efficiency.

The efficiency of the stabilizing effect of additives on PVC clearly depends on particle morphology. Ordered structures such as microspheres and rods are more effective than amorphous forms due to the combination of high specific surface area, structural barrier effects, and selective sorption of HCl. Specifically, TiPR rods, with their anisotropic morphology, create a “labyrinth effect” that slows the diffusion of oxygen and HCl. In contrast, amorphous TiP exhibits reduced protective efficiency due to its disordered structure. Small TiPMSI microspheres provide excellent protection and barrier performance owing to their large specific surface area; however, despite increasing the decomposition onset temperature, small TiPMSI microspheres stabilize less effectively than larger TiPMSII microspheres.

Analysis of the results indicates that the Ti/P ratio is not the determining factor in the stabilization efficiency of titanium phosphates. The stabilization provided by TiPMSI (Ti/P = 0.82) and TiPR (Ti/P = 0.86) is comparable despite their compositional differences. Therefore, hierarchically structured phosphates with developed surfaces, such as TiPMSI microspheres, are preferred for thermal stabilization of PVC as they offer a complex and effective protection mechanism.

## 4. Discussion

The proposed hierarchically structured titanium phosphate was synthesized by hydrothermal treatment of a titanium complex with mandelic acid in the presence of phosphoric acid at 15–20 °C for 2–3 h. This approach enables control over the morphology (size and shape of primary nanoparticles and their aggregates), achieves high crystallinity, and produces a material with a tailored high specific surface area and hierarchical structure, while employing relatively mild conditions and leveraging the structural potential of biomass (mandelic acid).

The obtained results of the study of the effectiveness of the stabilizing effect of modified titanium phosphates on the thermal stability of PVC compounds show that the developed surface and acidity of titanium phosphate allow for the effective binding of hydrogen chloride (HCl) released during the thermal destruction of PVC, which is an autocatalytic process. The HPO_4_^2−^/H_2_PO_4_^−^ groups bind HCl formed during the dehydrochlorination of PVC and suppress the autocatalytic process (Equations (1) and (2)):

HPO_4_^2−^ + HCl → H_2_PO_4_^−^ (1)

H_2_PO_4_^−^ + HCl → H_3_PO_4_ (2)

The hierarchical structure of the additives creates a physical barrier for the diffusion of oxygen and volatile decomposition products.

Excess acidic groups (H_2_PO_4_^−^) reduce thermal stability at the initial stage, which is expressed in a shift of the dehydrochlorination peak to a lower temperature region (Table 10).

Probably, in this case, the use of known synergistic additives of another type is promising. For example, epoxy compounds act through the following mechanism:

Epoxy group + HCl → Chlorohydrin

It is also known that secondary stabilizers can be used to suppress oxidation, blocking radical processes:

(1) Phosphites (P(OR)_3_) decompose hydroperoxides and interrupt chain reactions.
(2) Antioxidants (phenols, amines) stop radical processes.

However, the formation of a dense protective layer suppresses degradation at later stages, increasing the overall stability of PVC.

The optimal concentration of the additive is determined by the balance of processes occurring during heating. Excess acidic phosphate can reduce the onset temperature of dehydrochlorination, as observed in TGA/DSC data. Acidic groups (H_2_PO_4_^−^) at high concentrations exhibit weak acidity, which can catalyze the elimination of HCl at early stages. Subsequently, the formation of a dense protective layer during thermal decomposition creates a heat-resistant TiO_2_/phosphate coating on the PVC surface. This layer acts as a barrier to oxygen and heat diffusion, thereby slowing the release of volatile degradation products.

Titanium phosphate possesses high thermal stability, essential for PVC processing, and demonstrates a synergistic effect with traditional stabilizers such as calcium stearate and tribasic lead sulfate. Improved dispersion of structured forms (TiPMSI/TiPMSII microspheres, TiPR rods) is achieved due to the micro-scale size of aggregates (20–100 nm) and their hydrophilic surface. This results in a uniform distribution of TiP particles within the PVC matrix without agglomeration and establishes synergy with polar stabilizers (CaSt, TBLS) via hydrogen bonding and ionic interactions. The acid phosphate groups (≡P–O^−^ and HPO_4_^2−^) interact with stabilizer cations (Ca^2+^, Pb^2+^), forming coordinated complexes.

The synergistic binding of HCl by TiP and CaSt particles leads to an increase of about +28 °C in the onset temperature of sample weight loss when combined (TBLS + TiPMSI) (Table 5).

Amorphous TiP, being unstructured, tends to agglomerate, which diminishes the effectiveness of its combined action with CaSt/TBLS.

Therefore, TiP microspheres and rods are compatible with industrial stabilizers (CaSt, TBLS) owing to their hierarchical morphology, which ensures improved dispersion, chemical synergy, and reduced additive migration. In contrast, amorphous forms require surface modifiers to prevent agglomeration.

In summary, the use of hierarchical TiP in PVC composites is expected to significantly enhance the thermal stability of PVC relative to unstabilized polymer and potentially outperform some traditional stabilizer systems; improve initial coloration and maintain color stability under prolonged thermal aging; ensure effective dispersion in the PVC matrix due to the micro-scale aggregate size; reduce stabilizer migration; and provide a viable alternative to some heavy metal-based traditional stabilizers.

In an argon atmosphere, TiPMSI improves the thermal stability of the PVC composition, shifting the dehydrochlorination peak to 302 °C. However, in air, the additive reduces the fire resistance of the PVC composition by catalyzing the oxidation of the carbon residue. The mechanism of catalytic oxidation of carbon by TiPMSI particles in an oxidizing environment is governed by a combination of the chemical properties of the surface and their hierarchical structure. The key reasons in this case are:

1. Activation of oxygen on the surface of Ti^4+^ [46,50,51,52,53]. TI^4+^ ions in the TiPMSI structure act as active centers for o_2_ adsorption from the air. Intermediate complexes are formed:

Ti^4+^ + O_2_ → Ti^3+^ − O•^2−^ (superoxide radical) (3)

These radicals migrate along the particle surface due to their high mobility in the mesopores.

2. Decomposition of the polyene carbon skeleton [44,52,53,54,55]. Upon thermal degradation of PVC (>300 °C), polyene chains (--[CH=CH]_n_--) are formed. •O_2_^−^ radicals attack C=C double bonds, initiating chain oxidation:

Ti^3+^–O_2_•^−^ + >C=C< + 2H^+^ → Ti^4+^ + >C=O + H_2_O + >CH_2_ (4)

The formation of hydroxyl radicals (•OH) accelerates degradation by breaking C–C bonds.

3. The role of the hierarchical structure of TiPMSI [46,47,48]. The high specific surface area (>60 m^2^/g) increases the number of active Ti^4+^ centers. Mesopores (2–50 nm) facilitate the diffusion of O_2_ to the carbon skeleton. Nanoscale microspheres create extended interfaces where radical reactions are concentrated.

An experimental confirmation of this fact is a comparison with TiPR: TiPR crystal rods (without amorphous domains) do not exhibit catalytic activity.

In atmospheric air, the DSC curves do not show distinct minima at the initial stage of decomposition, unlike the DSC curves obtained in an argon atmosphere that correspond to the TG curves. The presence of oxygen significantly suppresses (masks) the characteristic endothermic peak of dehydrochlorination on the DSC curves. This phenomenon is explained by the competition between processes: exothermic oxidation reactions of the highly reactive polyene sequences formed during dehydrochlorination overlap with the endothermic HCl cleavage process, partially or completely compensating for its thermal effect, thereby causing a shift or disappearance of the characteristic peak.

It is important to note the two-stage nature of oxidation observed on the crystalline DSC curves as two consecutive exothermic peaks, which represent the main stages of the polymer’s thermal oxidative degradation. The first stage (400–450 °C) mainly corresponds to the reaction of alkyl radicals (R•) with oxygen to form peroxide radicals (ROO•) and their subsequent transformation into hydroperoxides (ROOH) through hydrogen abstraction:

RH + ROO• → ROOH + R•

The accumulation of ROOH is a relatively slow process.

The second, more intense stage (450–500 °C) is due to the thermal decomposition of accumulated hydroperoxides (ROOH → RO• + •OH), producing highly reactive alkoxyl (RO•) and hydroxyl (•OH) radicals. These radicals initiate rapid chain scission reactions (β-scission) and form volatile products, resulting in a significant mass loss accompanied by a strong exothermic effect. The decomposition of ROOH dramatically accelerates the overall oxidation process, acting as an autocatalytic step.

The catalytic activity of TiPMSI results from the redox properties of Ti^4+^ ions and the developed surface area. Hierarchical TiPMSI microspheres with a Ti/P ratio of 0.82 exhibit a dual function: they provide protection in an inert environment through HCl sorption and catalyze combustion in the presence of oxygen. Such additives are effective heat stabilizers for applications with limited oxygen access (e.g., cable insulation, sealed systems). However, in open conditions (e.g., building materials), TiPMSI microspheres require surface modification to suppress their catalytic activity.

Titanium phosphate TiPMSII enhances the thermal stability of PVC compositions in both inert and oxidizing atmospheres but shows greater effectiveness in argon. The hierarchical microsphere structure promotes uniform dispersion and the formation of a protective layer. For optimal thermal stability, it is recommended to use PVC compositions with TiPMSII under inert atmospheres or conditions that minimize oxidation.

Increasing the amount of titanium phosphate in PVC compositions has an ambiguous effect on thermal stability. All studied titanium phosphate modifications act as catalysts during the initial stage of PVC decomposition in oxidizing atmospheres, reducing the onset temperature and the maximum rate of dehydrochlorination. Nevertheless, titanium phosphates serve as effective fire retardants and carbon formers for PVC by significantly increasing the yield of heat-stable char, raising the onset temperature and maximum rate of oxidation of this char, and effectively suppressing the exothermic oxidation of carbon, especially in structured forms such as microspheres and rods. Notably, the amorphous sediment surpasses hierarchically structured particles (microspheres) in suppressing carbon oxidation efficiency.

Variations in the Ti/P ratio within the studied range are not a determining factor for thermal stability compared to the particle morphology.

Thus, the introduction of hierarchically structured titanium phosphates, particularly large TiPMSII microspheres or TiPR microrods, is an effective strategy to improve the thermo-oxidative stability and flame-retardant properties of plasticized PVC composites, primarily via condensed phase mechanisms (carbonization and barrier effects), despite their catalytic action during the initial degradation stage. Particle morphology plays a crucial role in their effectiveness.

A sufficient quantity of microspheres ensures excellent initial stability and forms a highly effective barrier against oxidation, leading to minimal mass loss during the secondary degradation stage and the absence of a pronounced exothermic peak. However, increasing the additive content beyond optimal levels, although enhancing initial stability, disrupts the formation of an ideal protective coke layer. This is due to possible particle agglomeration, which diminishes the effective surface area and barrier properties. Excess mineral phase renders the coke more porous and less continuous, intensifying oxidation processes in the second stage, as reflected by multiple DTG peaks, a strong exothermic DSC effect, and reduced residual mass.

The results further demonstrate that microsphere morphology and titanium phosphate with phosphorus predominance are highly effective, but an optimal concentration range must be maintained to achieve maximum protection across the entire temperature range. Exceeding this loading results in deteriorated high-temperature stability. Based on the findings and observed agglomeration mechanisms, optimal loading limits for titanium phosphates are proposed to prevent disruption of the coke protective layer (Table 11).

The following reasons justify this behavior. At high loadings (>3 wt. p. for microspheres), agglomeration occurs, and particles form clusters larger than 500 nm, disrupting the continuity of the coke layer. This results in a 40–60% decrease in the effective surface area, as confirmed by BET data. The critical percolation threshold for microspheres is approximately 2.8 wt. p. —the point at which a continuous protective layer forms. Exceeding this concentration leads to the development of pores ≥50 nm, which accelerates the diffusion of O_2_ and HCl.

Practical recommendations can be made for composites:

− For closed systems (cables, sealed products) TiPMSI: 2.0–2.5 wt. p. + 0.5 wt. p. CaSt;
− For open environments (building profiles): TiPR: 3.0–3.5 wt. p. + 1.0 wt. p. TBLS.

To optimize dispersion, surface modification is necessary. For example, silane treatment (APTES) of TiPMSI can reduce agglomeration by 50%. Additionally, La^3+^ ion exchange can be applied to TiPR to enhance its dispersion within the PVC matrix.

A loading limit of 3 wt. p. for structured titanium phosphate additives ensures a balance between barrier properties and the prevention of agglomeration. Exceeding this concentration necessitates surface modification of the particles.

## 5. Conclusions

Microstructured titanium phosphate is an effective heat stabilizer for PVC. Its use enables a significant increase in the thermal stability of the polymer during processing and enhances the long-term thermal stability of final products. Titanium phosphate is a promising alternative or additive to traditional stabilizers, particularly due to its potential environmental advantages. The effects of titanium phosphates (TiP) on the thermal degradation of PVC are summarized as follows:

1. The morphology of TiP particles plays a critical role in their stabilizing efficiency. Hierarchical structures—such as microspheres and rods—outperform amorphous TiP regardless of the Ti/P ratio (0.82–0.86). Key factors influencing performance include a high specific surface area (>60 m^2^/g) and structural orderliness. Microspheres provide an optimal balance by increasing the onset temperature of degradation, suppressing both the decomposition rate and exothermic processes, and ensuring a high residual carbon yield.
2. Dual role depending on the atmosphere: in an inert environment (argon), HCl binding (≡HPO_4_^2−^ + HCl → ≡H_2_PO_4_^−^) suppresses autocatalytic degradation, shifting the dehydrochlorination peak by approximately +15 °C on the DTG curve. In an oxidizing environment (air), Ti^4+^ ions catalyze oxidation of the carbon skeleton via the generation of superoxide radicals (-O_2_^−^), resulting in a decrease in Tmax by 6–10 °C.
3. Kinetic modifications are visible on the DTG curve. Peak consolidation occurs for TiPR rods, which create a labyrinth-like barrier, leading to a broad peak combining dehydrochlorination and oxidation stages. Conversely, TiPMSI microspheres induce radical generation, causing the DTG curve to split into multiple exothermic peaks above 400 °C.
4. Optimal additive concentrations are critical: for microspheres (TiPMSI), 1.5–2.5 wt. p. is recommended, as exceeding 3.0 wt. h. promotes particle agglomeration and disrupts the integrity of the protective coke layer; for rods (TiPR), 2.0–3.5 wt. p. provides the maximum barrier effect.
5. Practical recommendations include the following stabilizer loadings: for closed systems (e.g., cable insulation), TiPMSI combined with calcium stearate (CaSt) at 2.5 + 0.5 wt. p., respectively; for open systems (e.g., building materials), TiPR with tribasic lead sulfate (TBLS) at 3.0 + 1.0 wt. p., respectively. Introducing TiPMSI microspheres above 3 wt. p. in PVC formulations used in oxidizing atmospheres may catalyze oxidation reactions, diminishing thermal stability.
6. Surface modification techniques such as phosphorylation and silanization effectively suppress the catalytic activity of Ti^4+^ centers. This extends the applicability of titanium phosphate stabilizers to environments like building materials, where oxidation catalysis must be minimized.

In summary, microstructured titanium phosphates act as stabilizers based on combined chemical mechanisms (HCl binding) and physical effects (barrier formation). However, their effectiveness depends heavily on strict control of particle morphology and concentration to optimize performance and prevent adverse catalytic phenomena.

## Figures and Tables

**Table 1:** Main characteristics of modified titanium phosphates.

Sample	T, °C	Time, h	Starting Ti:P Ratio	Elemental Ratio Ti:P in the Precipitate
TiP	120	4	1/5	0.85
TiPMSI	180	4	1/8	0.82
TiPMSII	140	24	1/5	0.86
TiPR	120	72	1/4	0.86

where T, °C is the temperature of hydrothermal synthesis; Time, h is the time of hydrothermal synthesis.

**Table 2:** Thermal analysis data of PVC samples in an Ar atmosphere.

Sample	Ts, °C	Δm200 °C, %	Δm5%, °C	Δm10%, °C	m_f_, %	T1max DTG, °C	T2max DTG, °C	Tmax DSC, °C
1	2	3	4	5	6	7	8	9
PVC-p	162	0.7	239	255	1.53	302.7	440.5	299.2
PVC-p + TiP	127	2.7	213.5	240	2.25	304.1	447.3	296.7
PVC-p + TiPR	145	2.2	237	265	1.89	319.0	437.0	316.8
PVC-p + TiPMSI	155	1.0	244	260	2.15	302.0	446.8	297.7
PVC-p + TiPMSII	167	0.5	252	270	2.18	312.1	440.2	310.3

**Table 3:** DSC data for PVC samples in an Ar atmosphere.

№	ΔCp, J/(g∙K)	Tmax DSC, °C	ΔH, J/g	Interpretation
PVC-p	3.56	299.2	114.2	Standard dehydrochlorination
PVC-p + TiP	2.51	296.7	127.5	Weak barrier effect
PVC-p + TiPR	3.17	316.8	203.8	Suppression of exothermics
PVC-p + TiPMSI	4.35	297.7	121.7	Partial agglomeration
PVC-p + TiPMSII	2.63	310.3	142.0	Optimal barrier

**Table 4:** Thermal analysis data of PVC samples in an air atmosphere.

Sample	Ts, °C	Δm200 °C, %	Δm5%, °C	Δm10%, °C	m_f_, %	T1max DTG, °C	T2max DTG, °C	Tmax DSC, °C
1	2	3	4	5	6	7	8	9
PVC-p	157	3	216	246	1.50	301.1	438.0	441.3
PVC-p + TiP	114	2.4	213	239	2.25	301.1	437.4	443.1
PVC-p + TiPR	164	5.1	199	214	1.89	299.6	439.2	441.6
PVC-p + TiMSI	112	3.8	205	225	2.15	300.4	438.0	442.7
PVC-p + TiMSII	124	2.1	229	249	2.34	299.8	436.7	441.9

**Table 5:** DSC data of PVC samples in an air atmosphere.

№	ΔCp, J/(g∙K)	Tmax DSC, °C	ΔH, J/g	Interpretation
PVC-p	6.027	441.3	159.7	Sharp oxidation of carbon
PVC-p + TiP	4.097	443.1	173.7	ΔH growth—moderate stabilization
PVC-p + TiPR	5.057	441.6	196.4	High ΔH, barrier effect
PVC-p + TiPMSI	4.907	442.7	118.6	Catalysis of oxidation by microspheres
PVC-p + TiPMSII	5.951	441.9	138.2	Moderate suppression of oxidation

**Table 6:** Thermal analysis data of PVC samples with a high TiP content in an air atmosphere.

Sample	Ts, °C	Δm200 °C, %	Δm5%, °C	Δm10%, °C	m_f,_ %	Tmax DTG, °C	T2max DTG, °C	Tmax DSC, °C
1	2	3	4	5	6	7	8	9
PVC-p	157	3	216	246	1.50	301.1	438.0	441.3
PVC-p + TiP	134	1.4	226	243	11.07	292.4	441.9	444.9
PVC-p + TiPR	162	1.3	230	248	10.77	294.8	436.6	442.8
PVC-p + TiMSI	152	1.0	229	244	9.78	294.8	453.2	441.6
PVC-p + TiMSII	152	1.7	227	246	8.94	299.8	440.8	444.6

**Table 7:** DSC data of PVC samples with a high TiP content in an air atmosphere.

№	ΔCp, J/(g∙K)	Tmax DSC, °C	ΔH, J/g	Interpretation
PVC-p	6.027	441.3	159.7	Sharp oxidation of carbon
PVC-p + TiP	6.033	444.9	166.1	Growth of catalytic activity
PVC-p + TiPR	8.670	442.8	286.5	Preservation of barrier properties
PVC-p + TiPMSI	7.124	441.6	136.2	Preservation of the catalytic effect
PVC-p + TiPMSII	5.678	444.6	139.8	Violation of the coke layer

**Table 8:** Thermal analysis data of PVC samples with an integrated stabilizer in an air atmosphere.

Sample	Ts, °C	Δm200 °C, %	Δm5%, °C	Δm10%, °C	m_f,_ %	Tmax DTG, °C	T2max DTG, °C	Tmax DSC, °C
1	2	3	4	5	6	7	8	9
PVC-p	149	0.6	239	255	1.50	300.0	440.1	444.5
PVC-p + TiP	147	1.2	233	251	5.94	299.8	437.6	443.6
PVC-p + TiPR	139	0.6	243	260	2.43	306.1	441.5	446.7
PVC-p + TiMSI	177	2.0	238	256	5.61	301.7	438.3	444.5
PVC-p + TiMSII	154	0.5	246	262	2.64	307.3	441.0	446.2

**Table 9:** DSC data of PVC samples with an integrated stabilizer in an air atmosphere.

№	ΔCp, J/(g∙K)	Tmax DSC, °C	ΔH, J/g	Interpretation
PVC-p	5.338	441.3	143.8	Sharp oxidation of carbon
PVC-p + TiP	6.548	443.6	199.8	ΔH growth by 39%, synergy with TBLS
PVC-p + TiPR	4.608	446.7	180.0	Better thermal stability
PVC-p + TiPMSI	4.948	444.5	198.9	High efficiency
PVC-p + TiPMSII	5.629	446.2	170.8	Moderate improvement in stabilization

**Table 10:** Temperature at the beginning of the decrease in the mass of the sample (Ts, °C) for PVC compositions.

Sample	1 m. p. TiP + 5 m. p. CaSt, Ar	1 m. p. TiP + 5 m. p. CaSt, Air	5 m. p. TiP + 5 m. p. CaSt, Air	1 m. p. TiP + 1 m. p. CaSt + 4 m. p. TBLS, Air
PVC-p	162	157	157	149
PVC-p + TiP	127	114	134	147
PVC-p + TiPR	145	164	162	139
PVC-p + TiMSI	155	112	152	177
PVC-p + TiMSII	167	124	152	154

**Table 11:** Recommended ranges of titanium phosphate concentrations of various morphologies.

Morphology TiP	Optimal Loading	Limit Value	Performance Criteria
Amorphous TiP	0.5–1.0 wt. p.	≤1.5 wt. p.	Prevention of catalytic oxidation
Rod TiPR	2.0–3.5 wt. p.	≤4.0 wt. p.	Labyrinth barrier without pore closure
Microsphere TiPMSI/TiPMSII	1.5–2.5 wt. p.	≤3.0 wt. p.	Retention of dispersion without agglomeration, maximum Ts shift (+25–28 °C), minimum exothermic peaks (ΔH < 50 J/g)

## Data Availability

Dataset is available on request from the authors due to privacy.
